# Comparable performance on a spatial memory task in data collected in the lab and online

**DOI:** 10.1371/journal.pone.0259367

**Published:** 2021-11-29

**Authors:** Vladislava Segen, Marios Avraamides, Timothy Slattery, Giorgio Colombo, Jan Malte Wiener

**Affiliations:** 1 Aging and Dementia Research Center, Bournemouth University, Poole, United Kingdom; 2 Department of Psychology, Bournemouth University, Poole, United Kingdom; 3 German Center for Neurodegenerative Diseases, Magdeburg, Germany; 4 Department of Psychology, University of Cyprus, Nicosia, Cyprus; 5 CYENS Center of Excellence Nicosia, Nicosia, Cyprus; 6 ETH Zurich, Future Health Technologies, Singapore-ETH Center, Singapore, Singapore; Universidad de Chile, CHILE

## Abstract

Online data collection offers a wide range of benefits including access to larger and more diverse populations, together with a reduction in the experiment cycle. Here we compare performance in a spatial memory task, in which participants had to estimate object locations following viewpoint shifts, using data from a controlled lab-based setting and from an unsupervised online sample. We found that the data collected in a conventional laboratory setting and those collected online produced very similar results, although the online data was more variable with standard errors being about 10% larger than those of the data collected in the lab. Overall, our findings suggest that spatial memory studies using static images can be successfully carried out online with unsupervised samples. However, given the higher variability of the online data, it is recommended that the online sample size is increased to achieve similar standard errors to those obtained in the lab. For the current study and data processing procedures, this would require an online sample 25% larger than the lab sample.

## Introduction

In recent years, personal computers and the Internet have become widely accessible to most people, from a wide range of socio-economic backgrounds. This, together with the development of user-friendly experimental presentation platforms, such as Gorilla [1], Testable (testable.org), Pavlovia (pavlovia.org), JATOS (jatos.org), and MindProbe (mindprobe.eu), that support data collection from a wide range of devices (i.e. phones, tablets and computers), has led to increased popularity of online behavior data collection. In addition, recent widespread restrictions on in-person data collection because of the Covid-19 pandemic have encouraged many labs to consider switching to online research. The question we ask here is whether the data collected from online experiments are comparable to those obtained in the lab, at least in the context of spatial memory [2,3].

Running behavioral experiments online can offer many benefits including increased speed and reduced cost of collecting data [4]. This greatly reduces the time it takes from the initial theoretical conception of the experiment to obtaining the results. In addition, it frees up time that would have otherwise been spent on participant recruitment and testing, and allows researchers to focus on other tasks including data analysis, writing and experimental design. Furthermore, recruitment platforms such as Prolific and Amazon Turk enable access to very large samples of participants with diverse backgrounds (socio-economic status, age, ethnicity and education levels amongst many others) [5,6]. Access to such diverse populations can greatly improve the generalizability of results, compared to typical psychological experiments that often rely on testing biased samples (i.e. WEIRD populations) [7].

Despite the advantages that online data collection offers, researchers may be reluctant to move their research online due to the limited control that online experimentation offers, especially about the context in which the experiment is conducted. This may impact the quality of the data as both external and internal factors (e.g., noise, increased distraction, lower motivation etc.) may influence performance on the task. In addition, ensuring informed consent, explaining the task, and conducting effective debriefings online can be more difficult than in traditional laboratory settings. Thus, online experiments might require more thorough piloting of instructions, manipulations, and data-collection instruments [8,9]. Nevertheless, several studies have shown that online data collection does not compromise the data quality [10–16]. Yet, online data often contains more variability compared to data collected in the lab [6,15]. Such variability may either be introduced by reduced control over experimental settings, as highlighted above, or it may be an inherent property of studying more diverse participant groups, and therefore itself may be a variable of interest [17]. There are several steps that researchers can take to improve data quality and to reduce variability introduced by factors such as distractibility and lack of motivation (for a more comprehensive discussion see [9]). For example, asking participants how they solved the task and if they had cheated, has been shown to capture a wide range of potential outlier responses, including untruthful responses, distractions, and other unusual situations that may have compromised the quality of the data [18]. Using a progress bar and clear instructions also helps to increase motivation and engagement [18,19].

To explore the feasibility of online data collection, in the present study we implement a task from the field of spatial cognition. Research in spatial cognition has widely adopted virtual reality as it allows presenting realistic, yet highly controlled environments [20,21]. However, despite technological advances, there are important barriers in running complex virtual reality experiments online as they typically entail access to specific software and/or specialized hardware that most people are unlikely to have at home. Online testing may be suitable, however, for less technologically demanding tasks such as those used to study spatial memory and perspective taking [2,22–29]. Typically, these tasks present an encoding stimulus on the computer or on the paper, portraying a place or an array of objects that participants are asked to study. Then, a second stimulus is presented from a different perspective and participants are asked to judge whether the two stimuli depict the same place/array or not (i.e., to judge whether the objects in the array are in the same positions in the two stimuli). Such tasks use renderings of virtual environments or short videos and require same vs. different responses and can thus be implemented online with relative ease.

In this study, we assessed participants’ performance on a spatial memory task [30], in samples collected online and in the lab. The spatial memory task used in the current study investigates the precision with which object locations are remembered across different perspectives, an ability that supports navigation and orientation by allowing us to recognize previously- encountered places across different viewpoints [31,32]. Specifically, in this task participants viewed images of a virtual environment and memorized the position of the object. Following a brief delay, they were shown a new image of the same environment with the object removed, depicted from a different perspective and were asked to indicate as accurately as possible the object location. The main aim was to investigate whether data collected online would yield comparable results to data collected in person in the lab (for a more detailed discussion on the spatial memory task see [30]).

## Method

### Participants

The data from the lab-based version of the experiment were obtained from Segen et al., [30]. That study had 40 participants (Mean = 19.9, SD = 2.26, age-range = 18–31, 27 women and 13 men), individually tested in the laboratory. The online data were collected specifically for the investigation of differences between online and lab-based data collection. Forty different participants took part in the online variant of the experiment (Mean = 23.02, SD = 4.04, age-range = 18–33, 27 women and 13 men). All participants for the lab-based version were recruited through Bournemouth University’s participant recruitment system and received course credits for their participation. Data from the online sample were obtained either through Bournemouth University’s recruitment system, social media advertising and the online participant recruitment platform Prolific (https://www.prolific.co). All participants provided their informed consent in accordance with the Declaration of Helsinki [33].

### Materials

**Fig 1:**
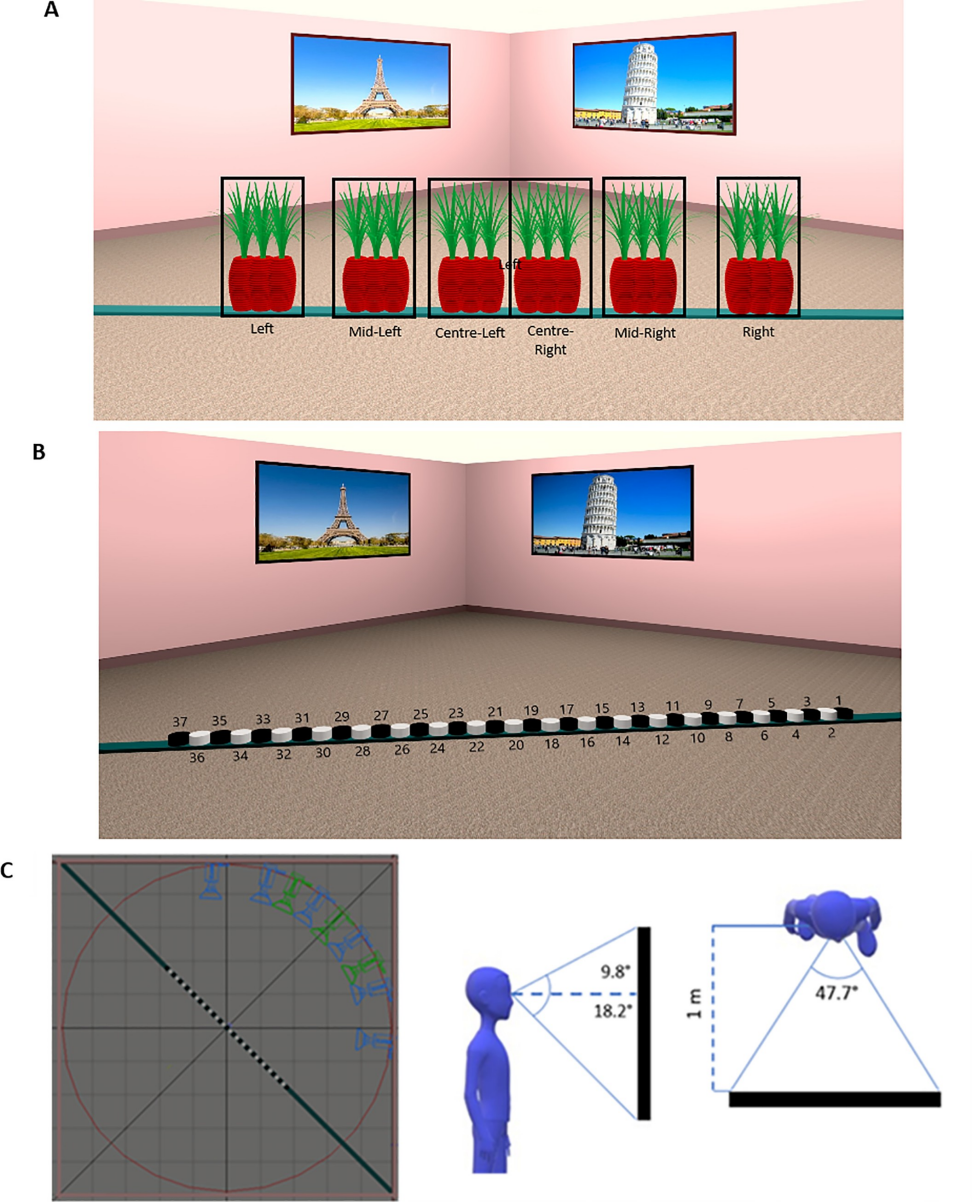
A Schematic of all possible Object Start Position groups; B Example of test stimuli; C Camera positions used to render encoding (green) and test (blue) stimuli.

The virtual environment was designed with 3DS Max 2018 (Autodesk) and consisted of a square 9.8m x 9.8m room. Posters depicting famous and easily recognizable landmarks were present on the walls of the room. The posters acted as distal cues that participants could rely on when encoding the location of the target object. Furthermore, a teal 14m-long plank was placed diagonally in the middle of the room (Fig 1A). In the encoding stimuli an object was placed on the plank in one of eighteen predefined positions (14, 28, 42, 84, 98, 112, 168, 182 and 192 cm either to the left or to the right of the center of the plank). For the purpose of the analysis, six groups with three object positions each were set up, e.g. object positions at 14, 28 and 42 cm to the left of the center were grouped together (Fig 1A). At test, the object was removed and 37 markers that marked all of the possible responses for the object position appeared (Fig 1B).

The experimental stimuli were renderings of the environment with a 60° horizontal field of view (FOV) and a 34° vertical FOV. A custom asymmetric viewing frustum that resembles natural vision with a 15% shift in the vertical field of view was used [34]. The cameras were arranged in a circle with a radius around an invisible diagonal line that was perpendicular to the plank. The encoding stimuli were rendered from three possible camera positions (Fig 1C). The test stimuli were rendered from a different viewpoint with a 30° perspective shift that resulted in a ≈ 2.5m camera displacement either to the left or to the right of the encoding viewpoint. In both encoding and test stimuli, the corner and a poster on each side of the corner were visible.

In the lab-based version stimuli were presented with OpenSesame 3.1.7 [35] on a monitor with 102cm diameter. Participants sat 1m away from the monitor and gave their responses using a standard computer keyboard. The keyboard was labeled such that a separate key corresponded to a specific position marker.

In the online version, the task required a laptop or desktop computer. Testable (https://www.testable.org) was used to present the stimuli, and participants responded by typing their responses into a text box. At the beginning of the experiment, participants were asked to adjust the screen zoom settings to ensure that the entire scene was visible during the experiment which was run in full-screen mode. Screen parameters including monitor size and resolution were not controlled in the online version of the task.

### Design

The study followed a mixed 6 (Object Position (OP): *Left*, *Mid-Left*, *Center-Left*, *Center-Right*, *Mid-Right* and Right) x 2 (Camera Direction: *Left/Right*) x 2 (Data Type: *Lab-Based/Online*) design. Data Type was a between subject factor and the rest were within-subject manipulations.

### Procedure

**Fig 2:**
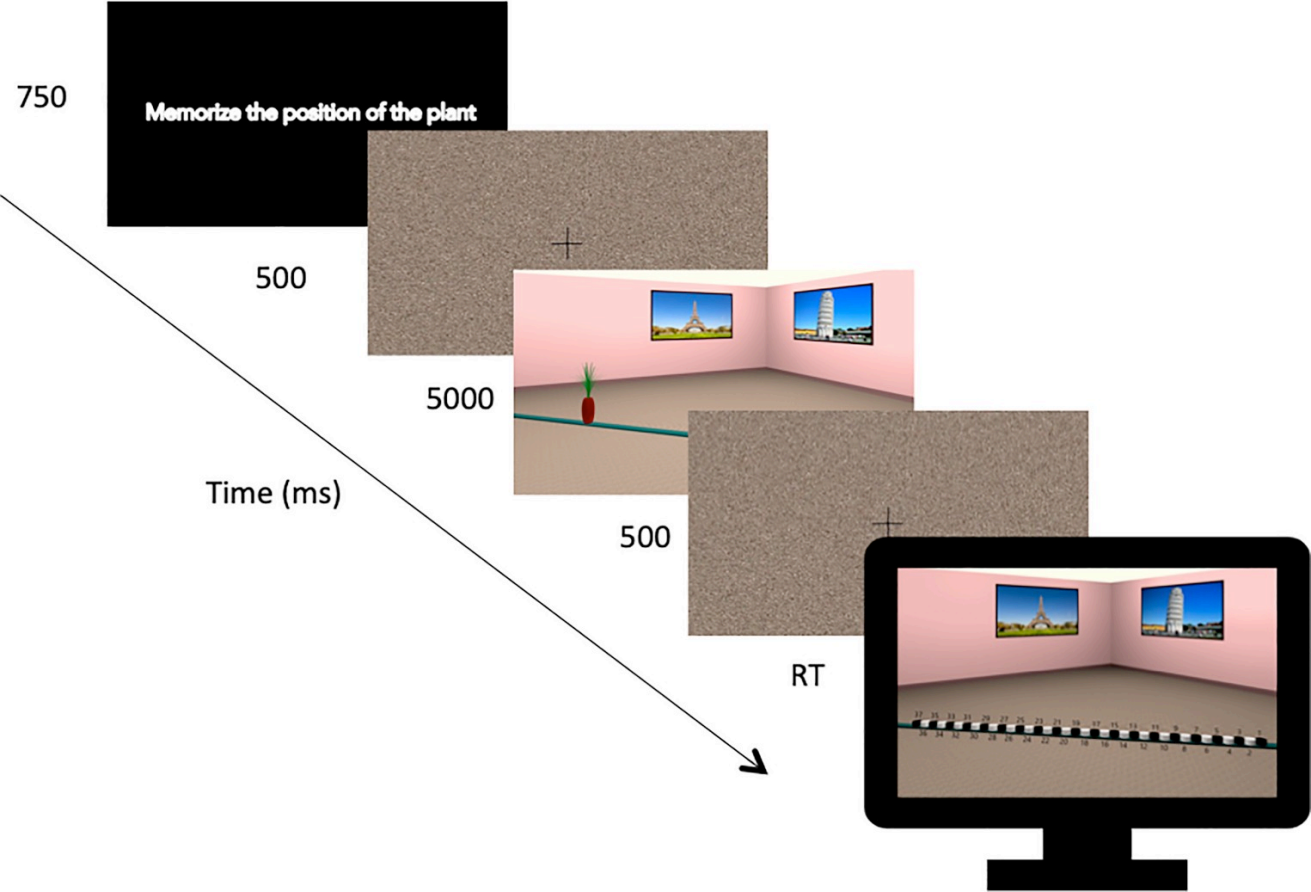
Experimental trial structure.

Each experimental trial started with a brief presentation of a display (750 msec) instructing participants to remember the location of the object. This was followed by the presentation of a fixation cross and a scrambled stimuli mask for 500 msec. Participants were then presented with the encoding display in which a rendering of the room was shown from one of three camera positions for five seconds. This rendering contained a plant occupying one of 18 possible positions. Following this encoding display, participants were again presented with a fixation cross and a scrambled stimuli mask for 500 msec. Then, participants were presented with the test display. This display depicted a rendering of the room following a 30° perspective shift in which the plant was removed, and 37 markers appeared (see Fig 2 for trial structure schematic). Participants were asked to choose one of the markers to indicate where they thought was the location in which the plant appeared in the encoding display. Responses were indicated either via a keypress or by typing the response using a text box in the lab-based and online versions, respectively. Both the lab and the online version of the task consisted of 108 experimental trials presented in randomized order with the experiment taking on average 30 minutes to complete.

## Results

Prior to analysis, outliers were removed from the individual absolute error distributions using the interquartile range method, for lab-based and online data separately. This resulted in 1.7% data loss for the lab-based data and 6.5% for the online data. The increased data loss in the online version of the task suggests that using a larger number of trials in online experiments is advantageous. For the data analysis Linear Mixed Effects (LME) models were run with the GLME4 package in R (R Studio). By-subject and by-item intercepts were included as random factors and fixed effects were effect coded.

### Absolute error

**Fig 3:**
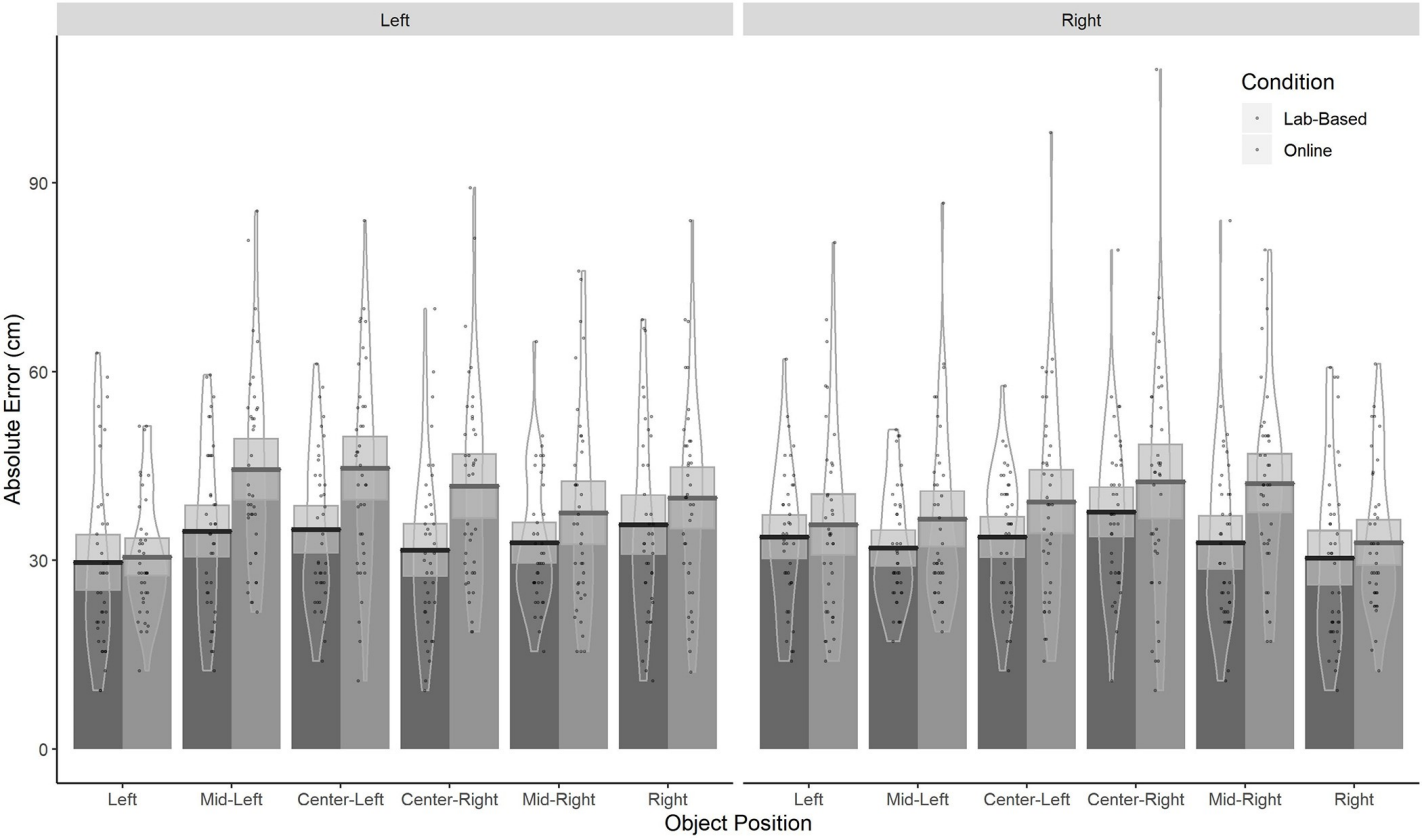
Absolute error as a function of Data Type, Object Position and Camera Direction.

Absolute error was computed by calculating the distance on the horizontal plane between the correct position and the position selected by the participant. Given the predefined arrangement of positional markers, minimum error could be 14 cm (unless participants select the correct position) and maximum error depended on the position of the object during encoding. Focusing on absolute (direction-free) errors, we ran an LME model with Data Type (*Lab-Based*/*Online*), Object Position (OP; *Left*, *Mid-Left*, *Center-Left*, *Center-Right*, *Mid-Right* and Right) and Camera Direction (*Left/Right*) as fixed factors. Results showed that absolute errors were larger in the *Online* data (Table 1 and Fig 3). However, the difference between the *Online* and *Lab* conditions was rather small (≈2.8cm) compared to the overall mean error/intercept (≈36cm). Thus, although significant, the overall increase in absolute error in the *Online* condition, compared to the *Lab*-*based* condition was less than 10% of the overall error. We also found that OP influenced absolute error, specifically, error was smaller for the *Left* OP when compared to the mean error across all OPs (with a trend for smaller error in the *Right* OP). Conversely, OPs closer to the center (*Center-Left* and *Center-Right*) yielded higher errors compared to the overall mean across all OPs. Notably, the influence of OP was greater in the *Online* data as the decline in errors in the *Left* OP (and a trend in the Right OP) was larger in the *Online* vs *Lab-Based* data. There was also an interaction between OP and Camera Direction with greater errors in situations when the camera moved further away from the object than when the camera moved closer to the object (i.e. greater errors for the *Right* OP when the camera moves left and smaller errors for the *Left* OP when the camera moves left). Interestingly, the reverse pattern was found for *Mid-Left* OP as errors were larger for left camera movements whilst smaller errors were found for *Center-Right* OP when the camera moved left.

**Table 1:** Coefficients from absolute error LME analysis.

	Absolute error (cm)		
*Predictors*	*Estimates*	*std*. *Error*	*t-value*
(Intercept)	36.027	1.037	**34.727**
Data Type (*Lab to Online*)	2.796	1.011	**2.766**
OP(*Right*)	-1.455	0.822	-1.769
OP(*Left*)	-3.574	0.822	**-4.346**
OP(*Mid-Left*)	0.805	0.820	0.982
OP(*Center-Left*)	1.932	0.823	**2.348**
OP(*Center-Right*)	2.044	0.824	**2.482**
Camera Direction (*Right to Left*)	0.323	0.368	0.880
Data Type (*Lab to Online*) * OP(*Right*)	-1.182	0.634	-1.863
Data Type (*Lab to Online*) * OP(*Left*)	-2.037	0.634	**-3.211**
Data Type (*Lab to Online*) * OP(*Mid-Left*)	0.763	0.631	1.209
Data Type (*Lab to Online*) * OP(*Center-Left*)	0.914	0.635	1.440
Data Type (*Lab to Online*) * OP(*Center-Right*)	0.816	0.636	1.282
Data Type (*Lab to Online*) * Camera Direction (*Right to Left*)	0.410	0.284	1.447
OP(*Right*) * Camera Direction (*Right to Left*)	2.633	0.822	**3.202**
OP(*Left*) * Camera Direction (*Right to Left*)	-2.646	0.822	**-3.218**
OP(*Mid*-*Left*) * Camera Direction (*Right to Left*)	2.300	0.820	**2.806**
OP(*Center*-*Left*) * Camera Direction (*Right*-*Left*)	1.314	0.823	1.598
OP(*Center*-*Right*) * Camera Direction (*Right to Left*)	-2.121	0.824	**-2.575**
Data Type (*Lab to Online*) * OP(*Right*) * Camera Direction (*Right to Left*)	-0.066	0.634	-0.104
Data Type (*Lab to Online*) * OP(Left) *Camera Direction (*Right to Left*)	-0.616	0.634	-0.971
Data Type (*Lab to Online*) * OP(*Mid*-*Left*) * Camera Direction (*Right to Left*)	0.853	0.631	1.352
Data Type (*Lab to Online*) * OP(*Center*-*Left*) * Camera Direction (*Right to Left*)	0.619	0.635	0.975
Data Type (*Lab to Online*) * OP(*Center-Right*) * Camera Direction (*Right to Left*)	0.874	0.636	1.374

### Signed error

**Fig 4:**
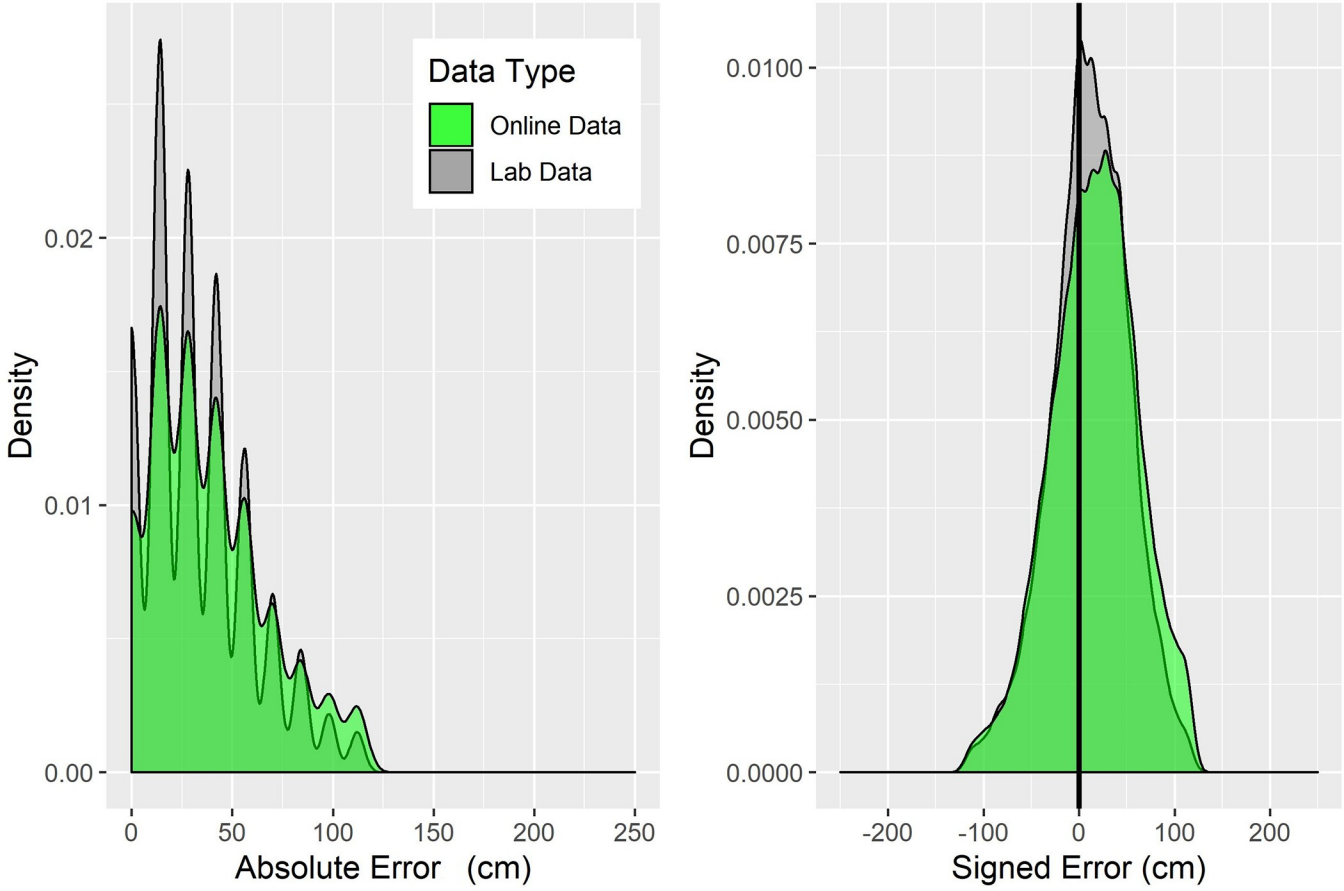
Distribution of absolute (left plot) and signed error (right plot) as a function of Data Type.

In a previous study [28], a different analysis of the lab-based data revealed a systematic bias, in that participants were more likely to make errors in the direction that is congruent with the camera movement between encoding and test, i.e. when the camera moved left participants were more likely to make errors to the left. To investigate if this bias is replicated in the online data, we estimated the magnitude of participants’ errors, similar to absolute error calculations. Additionally, we estimated the direction of the error, by coding errors to the left with a negative sign (i.e. -28cm) and errors to the right with a positive sign (i.e. 28cm). Next, we multiplied (folded) all of the errors where the camera movement was to the left by -1. Following this folding procedure, positive errors indicate errors in the direction congruent with the camera movement direction (i.e., camera moves to the left and participants make errors to the left) and negative errors indicate incongruent errors (i.e. camera moves left and participants make errors to the right), thereby allowing us to investigate the direction of the errors as a function of the Camera Direction. We ran an LME model with Data Type as a fixed factor. Overall, we found that participants’ errors were positive (intercept; β = 14.13, SE = 1.976, t = 7.149), with participants making errors congruent with the Camera Direction (Fig 4). Numerically, the magnitude of signed errors was larger in the Online compared to Lab-based condition (Fig 4). However, this difference did not reach statistical significance (β = 2.426, SE = 1.582, t = 1.534).

### Variance

In light of previous evidence that online data shows greater variance [6,9,11,15], we compared the variance in the absolute and in the signed errors in the Lab-based and Online conditions. To do so, we sampled with replacement across 10000 samples the variance for each error type separately in the *Lab-Based* and *Online* datasets, then compared these variances with a t-test. Variance was significantly larger in the *Online* than in the *Lab-based* data for both absolute error (*LabMean* = 678.17, *OnlineMean* = 819.77, p < .001) and signed error (*LabMean* = 1638.19, *OnlineMean* = 2035.17, p < .001) (Fig 4).

Given the increased variability observed in the online data, we estimated the sample size that would be required in the online variant of the task to achieve similar reliability, as indexed by the standard errors, of the Lab-based condition (Table 2) for both the absolute and signed errors. To do so, we calculated the standard error of the mean by dividing the standard deviation by the square root of the sample size, separately for the lab-based and online data (Table 2). Next, we estimated the sample size of the online data that would be required to render comparable standard error as that found in the lab-based given the standard deviation of the absolute and signed errors in the online sample. Results showed that 48.33 and 49.69 participants, respectively, would be needed to match the standard errors across the two conditions. Given that there were 40 participants in the original online sample, a 20.83% and a 24.23% increase in sample size would be needed to achieve comparable standard errors in the online data as in the lab-based data for absolute and signed errors, respectively. Consequently, in the current study, increasing the online sample size by around 25% would result in standard errors similar to those found in the lab-based data.

**Table 2:** Mean, standard deviation and standard error of absolute and signed error (cm).

	Absolute error (cm)			Signed error (cm)		
	*Mean*	*SD*	*SE*	*Mean*	*SD*	*SE*
Lab Data	33.08	26.05	4.12	11.58	40.48	6.40
Online Data	38.42	28.63	4.53	16.14	45.12	7.13

## Discussion

The aim of this study was to compare performance in a spatial memory task that was administered in a lab-based setting vs. online. Results show that absolute errors were slightly larger (less than 10% of the overall mean error) in the online variant of the study than in the typical lab-based setting. In a previous investigation of the lab-based data reported in the current study, we found that participants’ errors were biased in the same direction as the perspective shift at test [30]. Importantly, we showed here that this bias is also observed when the data are collected online. Although the bias was numerically larger in the online condition, the difference was not significant. We also found that for both absolute and signed errors, the variance was greater in the online data compared to the data collected in the lab.

Based on our previous research [29,36], we propose that the bias in the direction of the perspective shift is driven by (1) uncertainty about the position of the object following the perspective shift, caused by difficulties in understanding or perceiving the perspective shifts, and (2) difficulties in precisely encoding the position of the object. We believe that the egocentric spatial relations between the observer and the target object during encoding act as an anchor (c.f. Anchor and Adjustment Heuristics, [37]) that participants fail to adequately adjust after the perspective shift. As a result, they are more likely to make responses that are biased in the direction of the perspective shift (for a more detailed discussion see [30]). The presence of this perspective shift related bias in both the online and lab based versions of the task suggests that participants carried out the task using the same strategy regardless of whether they performed the experiment online or in the lab. This result suggests that this particular task is sensitive enough to produce the same patterns of findings in the lab and online, despite the increased variance in online testing.

Focusing on the more specific findings, it is clear that the position of objects during encoding impacted errors. Errors were smaller for objects that were placed further away from the center of the room (left and right object positions), whilst greater errors were found for object positions that were closer to the center of the room. Interestingly, the decrease in errors for the left and right object positions was larger in the online data. Lastly, absolute errors were larger in situations where the camera moved further away from the objects. A reverse pattern was observed for objects that were placed closer to the center. These effects were similar across the online and lab-based conditions.

Consistent with previous research [6,15], we found greater variance in the online sample compared to lab data. This is not surprising, as the context in which participants complete the experiment is likely to vary from one participant to the other in the online setting, e.g. different levels of background noise or presence of other distractions. In addition, some variance may be introduced by differences in the monitor sizes that are used by participants performing the study online as well as the distance between participants and their monitor. In the lab-based version, we controlled for monitor size and viewing distance as stimuli were presented on the same large monitor and participants sat 1 m away from this screen. Those conditions resulted in a closer match between the virtual environment and participants’ field of view. In the online version, those parameters were not controlled as no constraints on monitor sizes were introduced. Also, we did not instruct participants to adopt a particular viewing distance in the online condition. A potential avenue for future research would be to investigate whether controlling for these factors in the online version of the task leads to a reduction in variance in the online sample. Exploring these effects is facilitated by the developments in online experimental software that make it possible to automatically log screen details such as size and refresh rates.

In line with the less-controlled context in the online condition, more outliers were present in the online data set, which led to an exclusion of more data from the analysis (≈6.5%) compared to the lab-based condition (≈1.7%). Given that online data is expected to be more variable [9], it is not surprising that more outliers were present in the online condition and our findings are consistent with previous studies also showing that a larger proportion of data is excluded from online samples [11]. Given the greater amount of discarded data in the online condition, our recommendation for researchers wishing to collect data online is to increase the number of trials in their experiments from the lab-based equivalents.

Despite the larger variance and greater number of outliers, we found comparable patterns of results in the online and lab-based conditions, suggesting that the increased variance did not influence key effects, even with the same sample size of the lab-based study (i.e. 40 participants were tested in each condition). However, given that previous research indicates that effect sizes decrease in online variants of lab-based tasks [6], we suggest that larger sample sizes are used in online experiments. Specifically, based on our estimates, to achieve similar standard errors as those reported in the lab-based setting, we recommend that the online sample size be increased by 25% in tasks that resemble the one we used here. By increasing the online sample size to achieve comparable standard errors between the lab and online samples it is possible to, in part, compensate for the greater variability observed in this sample (which is likely to remain unchanged) by increasing the reliability of the estimates. However, we would like to note that the recommendation to increase the sample size by 25% should be treated with caution as it relies on the variability in the data (estimates are based on standard deviation) and therefore would be influenced by the data preprocessing strategies including outlier removal analysis undertaken by researchers as well as the variability in the dataset.

In closing, the current study provided evidence that online data collection can be successfully used to test spatial memory using static images. Although variance was greater in the online version of the task compared to the traditional lab-based version, the main pattern of results was replicated. Our conjecture is that similar paradigms that investigate spatial memory using static images can be successfully used to collect data online. However, we recommend increasing both the sample size and the number of trials to account for the increased variance observed with online testing.
